# Acoustofluidic separation enables early diagnosis of traumatic brain injury based on circulating exosomes

**DOI:** 10.1038/s41378-021-00244-3

**Published:** 2021-03-03

**Authors:** Zeyu Wang, Haichen Wang, Ryan Becker, Joseph Rufo, Shujie Yang, Brian E. Mace, Mengxi Wu, Jun Zou, Daniel T. Laskowitz, Tony Jun Huang

**Affiliations:** 1 Department of Mechanical Engineering and Materials Science, Duke University, Durham, NC 27708 USA; 2 Department of Neurology, Duke University, Durham, NC 27708 USA; 3 Department of Biomedical Engineering, Duke University, Durham, NC 27708 USA; 4 Department of Geriatrics, Duke University, Durham, NC 27708 USA; 5 Department of Electrical & Computer Engineering, Texas A&M University, College Station, TX 77840 USA

**Keywords:** Engineering, Microfluidics

## Abstract

Traumatic brain injury (TBI) is a global cause of morbidity and mortality. Initial management and risk stratification of patients with TBI is made difficult by the relative insensitivity of screening radiographic studies as well as by the absence of a widely available, noninvasive diagnostic biomarker. In particular, a blood-based biomarker assay could provide a quick and minimally invasive process to stratify risk and guide early management strategies in patients with mild TBI (mTBI). Analysis of circulating exosomes allows the potential for rapid and specific identification of tissue injury. By applying acoustofluidic exosome separation—which uses a combination of microfluidics and acoustics to separate bioparticles based on differences in size and acoustic properties—we successfully isolated exosomes from plasma samples obtained from mice after TBI. Acoustofluidic isolation eliminated interference from other blood components, making it possible to detect exosomal biomarkers for TBI via flow cytometry. Flow cytometry analysis indicated that exosomal biomarkers for TBI increase in the first 24 h following head trauma, indicating the potential of using circulating exosomes for the rapid diagnosis of TBI. Elevated levels of TBI biomarkers were only detected in the samples separated via acoustofluidics; no changes were observed in the analysis of the raw plasma sample. This finding demonstrated the necessity of sample purification prior to exosomal biomarker analysis. Since acoustofluidic exosome separation can easily be integrated with downstream analysis methods, it shows great potential for improving early diagnosis and treatment decisions associated with TBI.

## Introduction

Traumatic brain injury (TBI) represents a significant public health issue in the United States and internationally^1^. Globally, more than 50 million individuals are estimated to suffer from TBI annually, resulting in more than 50,000 deaths and 100,000 disabilities^2^. The complicated pathology and multiple forms of TBI result in unpredictable outcomes. Long-term complications from TBI—which include cognitive impairment, posttraumatic epilepsy, chronic traumatic encephalopathy, dementia, and cranial nerve injuries—can lead to lifelong consequences and high medical costs^3^. A major factor contributing to the progression of TBI to more serious conditions is a lack of early diagnostics for TBI. Approximately 40% of TBI patients fail to receive proper medical attention because of ineffective initial diagnosis^1,4^. Imaging modalities, which include computed tomography (CT) and magnetic resonance imaging (MRI), are sensitive to the presence of intracranial hemorrhage and edema. These modalities, however, cannot distinguish TBI cases that do not present significant structural injury^5^. Furthermore, there is a gap between primary screening based on symptoms and that based on imaging modalities. Many TBI patients with unclear or minor symptoms do not receive imaging-based diagnostics, resulting in inaccurate diagnoses^6^.

As an adjunctive approach for risk stratification in TBI, cerebrospinal fluid (CSF) is effective; however, the invasiveness of the procedure severely limits its utility^7^. To prevent high-risk individuals from suffering lifelong TBI-related disabilities or even fatal outcomes, it is critical to develop more effective noninvasive diagnostic methods for the early identification of tissue injuries^8^. This identification might facilitate triage and early decision-making in high-risk populations.

Analysis of biomarkers found in circulating exosomes represents a potential approach for low-cost and minimally invasive screening of TBI^9^. Exosomes are 30–150 nm membranous vesicles released by viable cells, including neurons, into bodily fluids. When cells are in abnormal states—including cells that have undergone malignant transformation, damage, or apoptosis-related changes—their secreted exosomes contain altered protein and molecular signatures^10^. Circulating exosomes found in the blood have been studied as potential targets for the development of blood-based point-of-care diagnostic platforms for cancer^11,12^. Because exosomes are released into circulation, they offer the potential to detect remote lesions. A similar strategy can be employed to monitor the status of the brain by analyzing exosomes released from injured CNS tissue into circulation.

Conventional attempts at using blood-based biomarkers for TBI diagnosis are restricted by the complex components of blood and the blood–brain barrier (BBB). These components often limit the ability to detect intrathecal injury markers in peripheral blood^13,14^. Exosomes, on the other hand, mediate intercellular communication and readily traverse the BBB^15^. Furthermore, the membranous vesicle structure of exosomes can protect disease-related biomarkers from degradation, so the biomarkers can remain in circulation longer. During neuronal injury and dysfunction events, neuron- and glial-secreted exosomes have been shown to contain biomarkers that can be released into peripheral blood, indicating the potential of assessing circulating exosomes for initial TBI diagnosis^16,17^. However, current exosome isolation technologies have low yields and can damage exosomes, resulting in a loss of biomarkers^18^. Furthermore, conventional ultracentrifugation and affinity capture take a long time (several hours to several days), which limits their use in point-of-care settings^19–22^. As a result, with most current exosome isolation technologies, it is not suitable to use exosomes as point-of-care biomarkers for TBI screening and diagnosis.

Here, we demonstrate an *acoustofluidic* (a combination of acoustics and microfluidics)^23,24^ device as a potential solution for exosome-based TBI diagnosis^25–28^. The underlying mechanism of acoustofluidics uses acoustic waves to generate acoustic radiation forces on particles in the fluid; the strength of the acoustic radiation force depends on the size and density of the particle^12,29–33^. The high biocompatibility of acoustofluidic devices also benefits downstream analysis by providing samples with complete structures and components^34–38^. Previously, we used acoustofluidics to separate bioparticles, including cells, bacteria, and platelets, in a highly biocompatible manner^39–41^. By generating surface acoustic waves (SAWs) with tilted-angle interdigital transducer (IDT) pairs, a tilted-angle standing wave field is generated and can separate particles based on differences in size and density^42–44^. In our previous study, an acoustofluidic device was used to successfully isolate exosomes from whole blood at a high yield while maintaining the morphology and molecular content of isolated exosomes^39,45^.

In this work, we further investigated whether the advantages of our acoustofluidic device can enable the early diagnosis of TBI based on biomarkers found in circulating exosomes. To simulate the neuropathology of TBI, we collected blood in a well-characterized rodent model of closed-head injury. By using acoustofluidics to isolate exosomes from mouse plasma samples and analyzing the abundance of glial fibrillary acidic protein (GFAP), we associated an exosomal biomarker for TBI with reactive astrocytes responding to brain injuries^7,46,47^. We found an increase in GFAP-positive exosomes soon after closed-head injury. Neuron intake experiments further showed that exosomes isolated from TBI mice had higher intake rates than exosomes isolated from healthy controls. These rates indicate that the isolated exosomes are bioactive and involved in TBI pathology, demonstrating acoustofluidic exosome separation technology as a powerful method for exosome-based TBI initial diagnosis and research.

## Results

### Working mechanism

**Fig. 1:**
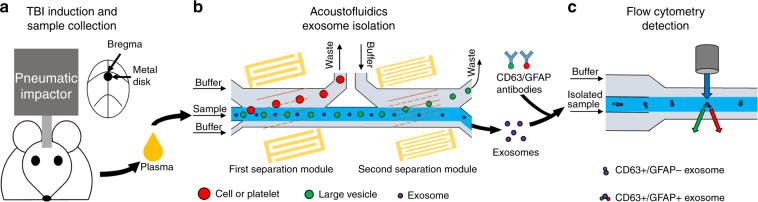
Schematics detailing the process of detecting exosomal biomarkers for TBI from the blood of animal models. **a** A pneumatic impactor induced TBI in a murine model; the location of impact was at bregma, covered by a metal disk. **b** Mouse plasma samples were processed through an acoustofluidic chip with two separation modules. Cells and platelets were removed by the first separation module, while large vesicles, including microvesicles and apoptotic bodies, were removed by the second separation module. Exosomes remained in the collected samples. **c** Isolated samples were stained with fluorescence-tagged anti-CD63 and anti-GFAP antibodies and analyzed with a flow cytometer to detect CD63+/GFAP+ events.

Early diagnosis is critical for the treatment of TBI to minimize secondary tissue injury and optimize triage and initial care. To evaluate the possibility of early TBI diagnosis based on the analysis of circulating exosomes, we used a murine head injury model to comprehensively simulate the pathological changes that occur immediately after TBI. Closed-skull impact models (Fig. 1a) are widely accepted as animal models of TBI because they recapitulate many of the clinical features associated with the condition, and they have been used to screen for molecular signatures of TBI in its early stages. Murine blood samples were collected before TBI treatment to establish baseline measurements of all blood components. Additional samples were collected at 3, 6, and 24 h after pneumatic impact to measure the levels of potential exosomal biomarkers for TBI.

Before flow cytometry analysis, samples were first processed by acoustofluidic exosome separation chips. When flowing through these devices, particles in the sample were separated based on differences in size. This separation eliminated larger cells, platelets, and other extracellular vesicles (such as apoptotic bodies and microvesicles), which are larger than exosomes. The driving force behind this size-based isolation was the acoustic radiation force that was induced by the acoustic fields generated by the IDTs around the channel (Fig. 1b). The radiation force on a particle is calculated by Eqs. 1 and 2:

1 $$F_r = - \left( {\frac{{\pi p_0^2V_p\beta _f}}{{2\lambda }}} \right)\phi \left( {\beta ,\rho } \right)\sin \left( {2kx} \right)$$

2 $$\phi \left( {\beta ,\rho } \right) = \frac{{5\rho _p - 2\rho _f}}{{2\rho _p + \rho _f}} - \frac{{\beta _p}}{{\beta _f}}$$

In these equations, the acoustic pressure is *p*_*0*_, the volume of the particle is *V*_*p*_, the wavelength is *λ*, the wavenumber is *k*, the distance from a pressure node is *x*, the density of the particle is *ρ*_*p*_, the density of the fluid is *ρ*_*f*_, the compressibility of the particle is *β*_*p*_, and the compressibility of the fluid is *β*_*f*_. According to these equations, larger particles experience a larger acoustic radiation force (F_r_), so they were more easily manipulated by the acoustic field and pushed to the waste outlet.

The device contained two separation modules to sequentially remove particles larger than exosomes. The IDTs in the first module generated a lower frequency acoustic field, which was optimized for removing cell components and platelets. It should be noted that the high-frequency acoustic field of the second separation module has a greater influence on micrometer-sized particles due to the larger acoustic radiation force they experience and can cause channel blocking if the larger particles are not removed first. Thus, the first separation module is required for eliminating large particles to ensure smooth operation of the device. The second module generated a higher frequency acoustic field for removing apoptotic bodies and microvesicles, which are extracellular vesicles larger than exosomes. Simulations of the acoustic pressure distribution and particle motion in the channel are shown in Fig. S1 in the Supporting Information, which illustrates how different particle sizes are directed as they flow through the pressure node lines generated by acoustic standing waves in the medium and travel to their appropriate waste and collection outlets.

### Validation of isolated exosomes

**Fig. 2:**
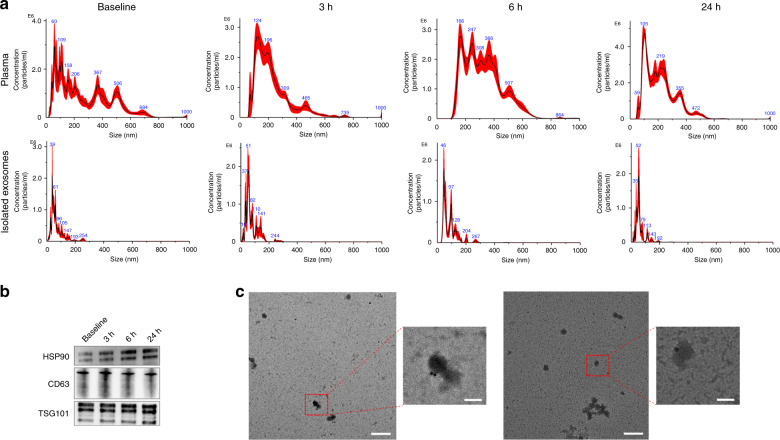
Validation of isolated samples. **a** Size distributions of unprocessed plasma samples and isolated exosome samples from the plasma collected before TBI and 3, 6, and 24 h after TBI treatment; the isolated samples mainly contained particles in the same size range as exosomes (30–150 nm). **b** Western blot analysis of the exosome biomarker proteins CD63, TSG101, and HSP90. Exosomal biomarkers were present in all isolated samples from plasma collected at different timepoints. **c** Immunotransmission electron microscopy of isolated exosome samples. Gold-conjugated antibodies are attached to the exosomes, indicating the presence of CD63 and GFAP proteins on the exosomes. Scale bars: 500 nm, 100 nm.

To identify the presence of normal exosomes—as well as exosomes related to TBI pathology—in the isolated samples, particle size distribution, general protein biomarkers for exosomes, and immuno-TEM morphological analysis were used for sample validation. For all samples, the isolated exosomes contained particles much smaller than the particles found in the unprocessed plasma samples. The size distribution of each sample indicated the successful removal of blood components larger than exosomes (Fig. 2a). The isolated samples were then assessed by western blotting (Fig. 2b). The presence of CD63, TSG101, and HSP90, which are general exosomal protein biomarkers, showed that the isolated samples contained exosome components. To further validate the morphology and detect the presence of CD63 and GFAP on the surface of the isolated exosomes, the samples were stained with anti-CD63 or anti-GFAP primary antibodies followed by gold-conjugated secondary antibodies. While CD63 is a general exosomal marker, GFAP is associated with astrocyte activation in response to injury and is considered a biomarker related to the pathology of TBI. The TEM images showed vesicle structures with gold nanoparticles attached (Fig. 2c). These results demonstrate that the isolated samples contained exosome-like vesicles with CD63 or GFAP.

### Astrocyte TBI exosome intake

**Fig. 3:**
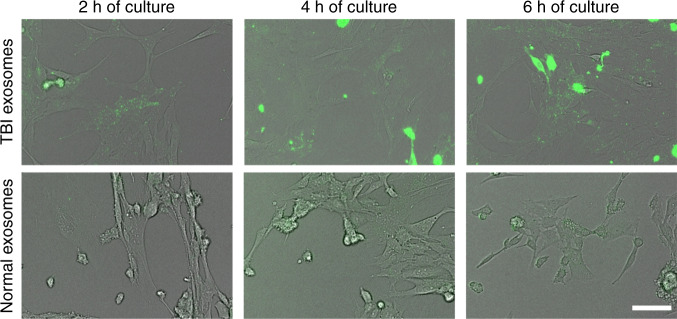
C8-D1A astrocytes cultured with isolated exosomes from TBI mouse plasma were compared with exosomes isolated from mouse plasma before TBI treatment. Exosomes isolated from plasma collected 24 h after TBI treatment had a higher intake rate by astrocytes, as demonstrated by fluorescence intensity, indicating the existence of exosomes that participate in neuronal activities. Scale bar: 50 μm.

To evaluate whether the detected CD63+/GFAP+ exosomes were indicative of TBI pathology, isolated samples pre-TBI and 24 h after TBI were tagged with DIO fluorescent dyes and added to C8-D1A cultured astrocytes. During the 6-h intake period, the exosomes isolated 24 h after TBI (TBI exosomes) showed greater intracellular fluorescence than the exosomes isolated before TBI (normal exosomes) (Fig. 3). This result suggests that in the TBI samples, there were more exosome populations that could be internalized by astrocytes. Since exosomes participate in intercellular communication and are transported among neurons in neurodegenerative diseases, the high intake of exosomes from the samples collected 24 h after TBI indicates that the pathological functions of TBI exosomes were preserved after acoustofluidic isolation.

### Feasibility of TBI exosomal biomarker detection using flow cytometry

**Fig. 4:**
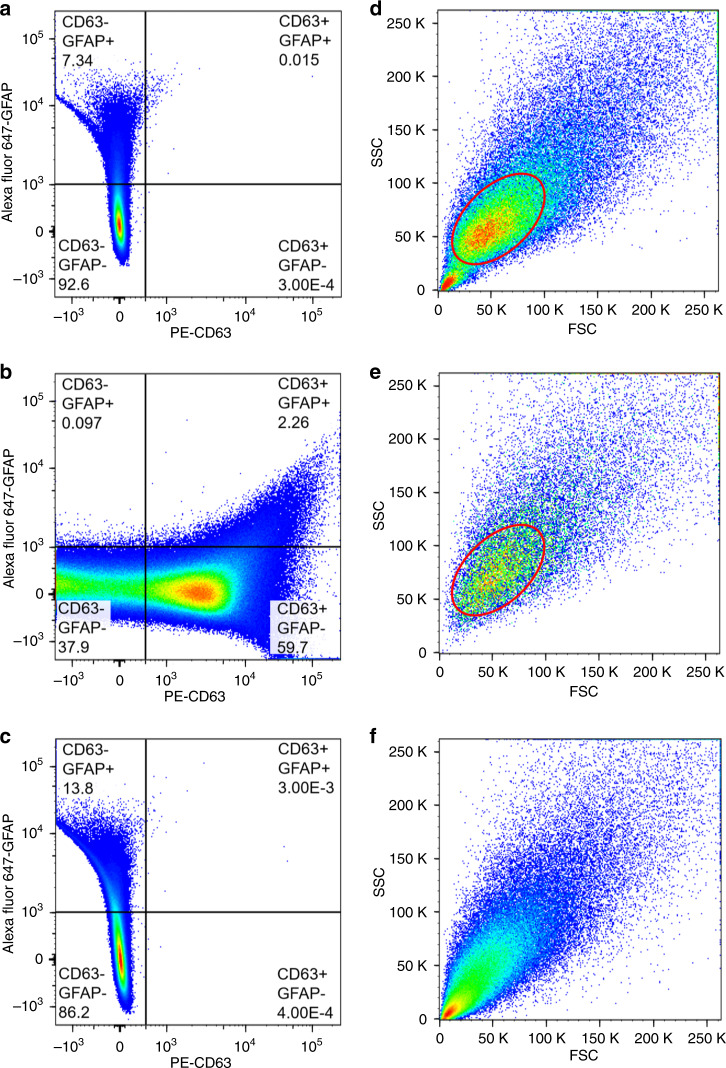
Single staining of exosomes isolated from plasma collected 24 h after murine TBI treatment. **a** Exosomes without fluorescence-tagged antibody staining. Most of the events (92.6% of a total of 100,000 events) were concentrated in the CD63−/GFAP− area. **b** Exosomes stained only with PE-CD63. The events migrated to the CD63+/GFAP- area (59.7% of the total events were moved to the CD63+/GFAP− dimension, compared with 0.03% in Fig. 4A); these results represented the presence of normal exosomes containing the CD63 protein. **c** Exosomes stained only with Alexa fluor 647-GFAP. The events in the CD63−/GFAP+ area showed a percentage increase compared with that in Fig. 4A (13.8% of events were distributed to the CD63−/GFAP+ dimension, compared with 7.34% in Fig. 4A), indicating the existence of TBI exosomes containing the GFAP protein. **d** FSC and SSC of the CD63−/GFAP+ area of Fig. 4A, indicating that events were induced by noise from large particles, as shown by the red circle. **e** FSC and SSC of the CD63+/GFAP+ area of Fig. 4B indicated that most events were induced by noise from large particles. **f** FSC and SSC of the CD63−/GFAP+ area of Fig. 4C indicated that most events were from small particles, including exosomes.

Currently, using a flow cytometer for exosome detection is challenging due to the small size of exosomes and a lack of standard operating procedures. To increase the ability of the flow cytometer to detect exosomes, fluorescent GFAP antibodies and CD63 antibodies were used to tag exosomes that participated in the pathology of TBI and normal exosomes, respectively. To determine the detection thresholds for distinguishing between CD63−/CD63+ particles and GFAP−/GFAP+ particles, exosomes isolated from mice 24 h after TBI treatment were separated into three samples of identical volume and stained separately with fluorescent GFAP antibodies and CD63 antibodies. The remaining sample was not stained and was used to obtain the baseline fluorescence strength measurements. Compared with that of the nonfluorescently tagged exosomes, the fluorescence signal of PE-CD63-tagged exosomes rapidly increased (Fig. 4a, b). Most of the events in the nonfluorescently tagged sample were concentrated in the CD63−/GFAP− area. For the PE-CD63-tagged samples, some of the events migrated to the CD63+/GFAP− area. This signal difference showed that fluorescent antibodies could stain CD63+ exosomes, and exosomal CD63 fluorescent signals could be detected by a flow cytometer. The event distribution comparison between nonfluorescently tagged exosomes and Alexa fluor 647-GFAP tagged exosomes was insignificant. Compared with GFAP− normal exosomes, TBI pathology-related GFAP+ exosomes had a much lower population that did not show obvious event migration. However, the particle size (FSC) and granularity (SSC) information showed that the particles in the GFAP+ area of nonfluorescently tagged samples contained events from large and granular particles, as shown by the red circle (Fig. 4d). In the GFAP+ area of the Alexa fluor 647-GFAP tagged samples, most of the events were from smaller, less granular particles (Fig. 4f). This size and granularity distribution difference indicated that the GFAP+ events in the nonfluorescently tagged sample were noise, while the majority of GFAP+ events in the Alexa fluor 647-GFAP-tagged sample were from exosomes containing GFAP. Furthermore, the Alexa fluor 647-GFAP-tagged samples had more events containing GFAP+ signals (13.8%) than the nonfluorescently tagged sample (7.34%), indicating that GFAP+ exosomes could be stained and detected (Fig. 4a, c). The GFAP+ events in the PE-CD63-tagged samples showed FSC and SSC distributions similar to those of the nonfluorescently tagged samples. This result is shown by the red circle and indicated that these events were also caused by noise (Fig. 4e).

### TBI-related exosome population change after TBI treatment

**Fig. 5:**
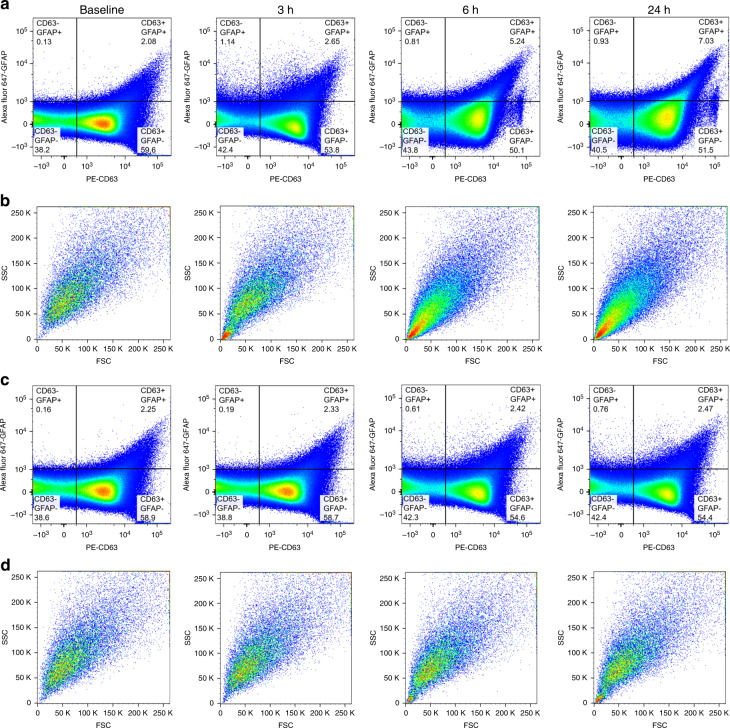
CD63+/GFAP− exosome population change after murine TBI treatment. **a** Acoustofluidic-isolated exosomes stained with both PE-CD63 and Alexa fluor 647-GFAP. Samples collected at different times showed that CD63+/GFAP+ events increased after TBI (events in the CD63+/GFAP+ dimension increased from 2.08% of baseline to 2.65%, 5.24%, and 7.03% at 3, 6, and 24 h post-TBI treatment, respectively), indicating that the number of circulating TBI-related exosomes increased immediately following TBI. **b** FSC and SSC of the CD63+/GFAP+ area for exosomes at each timepoint. These results suggest that while the CD63+/GFAP+ events were initially the result of noise from larger particles, the events detected at later timepoints after TBI treatment were due to exosomes. **c** Direct flow cytometry analysis using plasma samples did not demonstrate an increase in CD63+/GFAP+ events after TBI (the events in the CD63+/GFAP+ dimension were 2.25%, 2.33%, 2.42%, and 2.47% at baseline and at 3, 6, and 24 h, respectively; these results show no increasing trends). **d** FSC and SSC of the CD63+/GFAP+ area of plasma collected at each timepoint. The majority of the signals are due to noise from large particles.

Based on the threshold for distinguishing CD63+/− and GFAP+/− events, plasma samples from three TBI mice were processed via acoustofluidic isolation and flow cytometry analysis. Samples were collected before TBI treatment and 3, 6, and 24 h after treatment. The CD63+/GFAP+ events increased with time after treatment (Fig. 5a). The CD63+/GFAP+ events increased from 2.08% of baseline to 2.65%, 5.24%, and 7.03% at 3, 6, and 24 h after TBI, respectively, indicating that the number of circulating CD63+/GFAP+ exosomes increased. To evaluate the percentage of noise signals in the CD63+/GFAP+ events, the FSC and SSC data of these events were measured (Fig. 5b). The particle size and granularity of CD63+/GFAP+ events detected for the sample collected before TBI were significantly larger than those of exosomes, indicating that these events were induced by noise from large particles. For the sample collected 3 h following TBI, there was a population increase of CD63+/GFAP+ events induced by low FSC/SSC signals, and this population continued to increase at 6 h and 24 h. This CD63+/GFAP+ population size change indicated that before TBI, the CD63+/GFAP+ signals were noise and that TBI-induced exosomes were released into the blood after TBI treatment. This increase in CD63+/GFAP+ exosomes after TBI could only be identified in the acoustofluidic-isolated samples; the flow cytometry results of unprocessed plasma samples did not show an increase in the number of CD63+/GFAP+ events (Fig. 5c). The FSC and SSC results showed that the CD63+/GFAP+ events in plasma were mostly induced by large particles, indicating the need for acoustofluidic separation to remove larger particles prior to flow cytometry analysis (Fig. 5d).

**Fig. 6:**
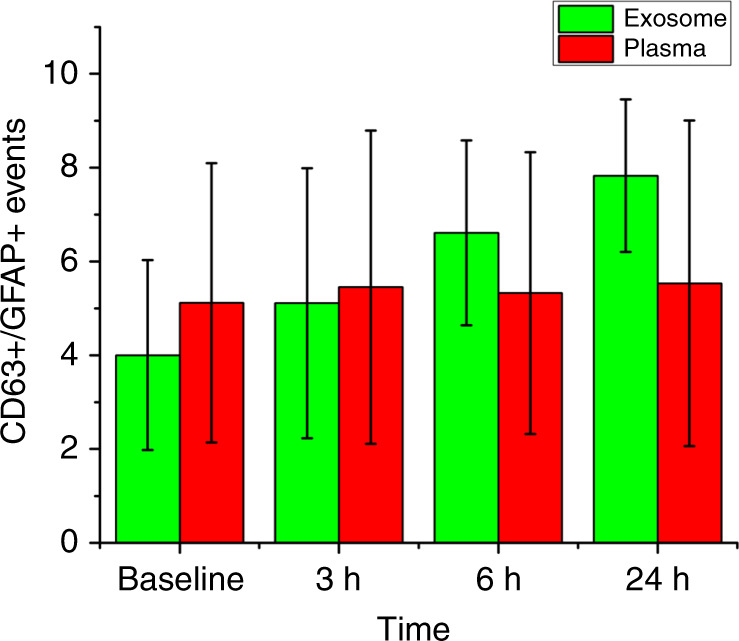
Comparison of CD63+/GFAP+ events in plasma samples and exosome samples separated via acoustofluidics. CD63+/GFAP+ events in isolated exosomes increased, while plasma samples showed no change with time after TBI, indicating that acoustofluidic separation has the potential to reveal TBI-related blood exosome population changes.

Acoustofluidic-isolated exosomes from the other two mice showed similar trends: the number of CD63+/GFAP+ events increased following TBI treatment, and we were unable to detect a similar trend using unprocessed plasma samples (Fig. S1, S2). Only acoustofluidic-isolated samples showed an increase in the number of TBI-related exosomes after TBI, indicating that the acoustofluidic technology had a positive effect on downstream analysis (Fig. 6). To evaluate whether the difference between baseline CD63+/GFAP+ event counts and those observed in each sample group were significant, we conducted a one-way analysis of variance (ANOVA). According to the ANOVA, significant differences in exosome levels were observed between the baseline and 24 h post-TBI treatment groups (*p*-value = 0.032, which is less than 0.05). Additionally, no significant differences in exosome levels were observed between the baseline and 24 h groups in the original samples (*p*-value = 0.883, which is larger than 0.05), suggesting that TBI-relevant exosomes present in blood plasma cannot be detected without acoustofluidic exosome isolation.

## Discussion

By applying acoustofluidics, we were able to successfully separate TBI-related exosomes from blood plasma, as detected via flow cytometry. Animal experiments indicated that after TBI treatment, the number of GFAP+/CD63+ exosomes in plasma increased over time, which suggests a potential role for exosomes as biomarkers for early screening and medical decision-making in patients with TBI. Exosomes might also be a tool to monitor the efficacy of interventions and individualize therapy as well as serve as surrogate measures of acute brain injury in clinical trials. According to comparative tests using unprocessed plasma, an increase in GFAP+/CD63+ exosomes was not detected, suggesting that acoustofluidic separation played an important role in sample processing for exosome analysis. One potential explanation for the importance of using acoustofluidic separation prior to flow cytometry analysis is that it can eliminate larger particles that induce noise and thus interfere with the ability to detect exosomes. Because of the small size of exosomes (30–150 nm), using flow cytometry for exosome detection is challenging. Small vesicle structures may not generate strong enough forward-scattered light (FSC) and side-scattered light (SSC) signals, which are used as indicators of the size and internal complexity of a particle, respectively. Smaller particles also suggest that there are few biomarkers and low fluorescence from antibodies with specifically labeled exosomes. Although increasing the laser strength and decreasing the detection threshold can enable the detection of FSC, SSC, and fluorescent signals from exosomes, it also increases background noise and unwanted signals from other large particles, including microvesicles, apoptotic bodies, and platelets. Acoustofluidic separation decreases interference from large blood components, which enables the successful monitoring of TBI-related exosomes in blood. Because of the high laser intensity, large instrument size, and limited availability, a flow cytometer is perhaps not the best approach for exosome detection in practical settings. However, due to its high sensitivity, this method was excellent for device characterization. Our results suggest that acoustofluidic separation is essential for sensitive exosome detection. We envision that a miniaturized exosome detection unit, through electrochemistry, acoustics, or optics, can be integrated with our acoustofluidic exosome separation unit to enable an all-in-one exosome separation and detection device that will be invaluable in the field of TBI diagnosis.

Although we used plasma samples in this study, this two-module acoustofluidic device can continuously isolate exosomes from whole blood. Blood plasma was selected as the candidate biofluid for exosome separation in this study due to its more convenient sample storage and transportation requirements compared with those of whole blood, as the plasma samples can be frozen. However, we have demonstrated previously that our acoustofluidic separation device is effective in the removal of all cells present in whole blood and could easily be used to isolate exosomes from whole blood samples^48,49^. In addition, our device can be integrated with flow cytometry-like detection devices. A combination of an acoustofluidic separation unit with an optical detection unit has potential as an easy-to-operate, biocompatible, and rapid TBI early diagnosis system. Developing a point-of-care diagnostic test for TBI would guide triage and early management decisions in patients with TBI, potentially improving their outcome. Compared with conventional exosome isolation methods, including ultracentrifugation and antibody capture, acoustofluidic separation is more rapid, biocompatible, efficient (higher yield), low-cost, and much easier to handle and integrate with downstream analysis. Moreover, this approach may be used to identify new therapeutic targets designed to reduce excitotoxicity, neuroinflammation, and secondary tissue injury due to intracranial hypertension, as well as serve as a clinical surrogate in early clinical trials.

## Materials and methods

### Device fabrication

Fabrication of the acoustofluidic device followed our previously published procedures^48^. During the photolithography and lift-off processes, two pairs of tilted-angle IDTs with frequencies of 20 and 40 MHz were deposited on a Y+128° X-propagation lithium niobate (LiNbO3) substrate. The designs of the IDTs were created by UV exposure combined with SPR3012 photoresist (MicroChem Corp., USA) aligned by an MA/BA6 mask aligner (SUSS MicroTec., Germany). Unwanted photoresist was then removed by CD26 developing solution (MicroChem Corp., USA), and a double metal layer (Cr/Au, 50 Å/500 Å) was deposited on the substrate by an e-beam evaporator (Semicore Corp., USA). IDTs with electrode widths of 50 and 25 μm were formed through a lift-off process using a PRS3000 resist stripper (VWR, USA). A silicon mold was created by SU-8 photoresist (MicroChem Corp., USA) through soft lithography. A PDMS microchannel with a height of 100 µm and width of 800 µm was solidified by silicone elastomer curing reagents (Dow Corning, USA) on the mold. The substrate and microchannel were then treated by oxygen plasma coating and bonded at 65 °C overnight.

### TBI animal model and plasma collection

The murine closed-head injury model used in this study was previously described, and it is associated with reproducible histological and functional deficits^50^. As is the case with human TBI, selective closed-head impact injures vulnerable neurons in the cortex and hippocampus of the mouse. This impact is associated with vestibulomotor deficits and long-term neurocognitive deficits. Although animals lose body weight, they rapidly regain spontaneous ventilation, righting reflex, and the ability to ambulate.

Briefly, 12–14-week-old C57Bl/6 J male mice (Jackson Laboratories, USA) were endotracheally intubated after anesthesia induction with 4.6% isoflurane, and lung ventilation was achieved with 1.6% isoflurane in 30% O_2_/70% N_2_ through tracheal intubation. A rectal probe was used to maintain mouse core body temperature at 37 °C. After the mouse was secured in a prone position, the head was shaved to identify anatomical landmarks. The skull was then adhered to a concave 3 mm metallic disc just caudal to bregma, and a single midline impact was delivered to the center of the disc through a pneumatic impactor (Air-Power Inc., USA). The isoflurane was reduced to 0%, the ventilation was disconnected, and the trachea was extubated after recovery of spontaneous respiration. Each mouse was then returned to its home cage with free access to food and water. The blood extraction timepoints were before impact and 3, 6, and 24 h after impact. For each timepoint, 200 µL of whole blood was extracted with a heparinized syringe through the thoracentesis and ventricle and then centrifuged at 2000 × *g* for 10 min to eliminate blood cells. Collected plasma samples were stored at −80 °C before acoustofluidic exosome isolation.

### Experimental settings for exosome separation

A syringe pump (NeMESYS, CETONI GmbH, Germany) was used to independently control the sample and the PBS (Thermo Fisher, USA) sheath flow. To avoid excessive heat generation from the IDT, a Peltier cooling system (TEC1-12730, Hebei IT, China) powered by a variable DC power supply (TP1505D, Tekpower, USA) was used for cooling the acoustofluidic chip during exosome separation. The separation process was monitored with an upright microscope (BX51WI, Olympus, Japan) combined with a CCD camera (CoolSNAP HQ2, Photometrics, USA). A function generator (E4422B, Agilent, USA) and an amplifier (100A250A, Amplifier Research, USA) were used to power the IDTs and generate SAWs. Isolated exosome samples were collected in 1.5 mL centrifugation tubes placed on ice, and the size distributions of particles were measured with a nanoparticle tracking analysis (NTA, Nanosight LM10, Malvern, England) system.

### Immunoblot

A mixture of Pierce Cell Lysis Buffer (Thermo Fisher, USA) and Halt Protease Inhibitor Cocktail (Thermo Fisher, USA) was used to lyse exosome samples. The samples were then processed by electrophoresis and transferred to a polyvinylidene fluoride membrane (Bio-Rad, USA). Mouse anti-CD63 (sc-5275, 1 μg/mL, Santa Cruz, USA), mouse anti-HSP90 (ab13492, 1 μg/mL, Abcam, USA), and rabbit anti-TSG101 (ab30871, 1 μg/mL, Abcam, USA) were used as primary antibodies for incubating the membrane at 4 °C overnight. Goat anti-mouse IgG (ab97040, 0.05 μg/mL, Abcam, USA) and goat anti-rabbit IgG (ab97080, 0.05 μg/mL, Abcam, USA) were used as secondary antibodies. Protein abundances were analyzed with a ChemiDoc XRS+ system (Bio-Rad, USA).

### Exosome cell intake rate

C8-D1A astrocytes, which are an astrocyte cell line generated from the cerebral cortices of neonatal C57BL/6 mice, were cultured in Dulbecco’s Modified Eagle’s Medium (Gibco, USA) supplemented with 10% exosome-depleted fetal bovine serum (Gibco, USA). Isolated blood exosomes from the same mouse before pneumatic impaction TBI treatment and 24 h after TBI treatment were labeled with fluorescent tracking dye (Invitrogen, USA) and were separately added to C8-D1A culture medium. C8-D1A cells were observed with a ZOE Fluorescent Cell Imager (Bio-Rad, USA) at 2, 4, and 6 h after adding isolated exosomes to evaluate exosomal intake rates by fluorescence strength.

### Immunotransmission electron microscopy

For antibody-conjugated gold staining, 10 μL of isolated exosome sample was covered by a copper grid film (Electron Microscopy Sciences, USA) for 20 min of absorption. The grids were then incubated with a 100 μL drop of 50 mM glycine PBS solution for 10 min, followed by PBS washing. 1% BSA PBS solution was then used for blocking grids. Primary antibodies—mouse anti-CD63 (NBP2-32829, Novus Bio., USA) and rabbit anti-GFAP (NB300-141, Novus Bio., USA)—were independently used to stain the grids for 30 min, followed by PBS washing. The grids were stained with 10 nm gold-conjugated goat anti-rabbit IgG (Electron Microscopy Sciences, USA) and 15 nm gold-conjugated goat anti-mouse IgG (Electron Microscopy Sciences, USA) secondary antibodies for 30 min and then washed with PBS. The grids were then incubated with uranyl–acetate solution for 10 min followed by water washing for complete negative staining. An electron microscope (FEI Company, USA) was used to image the grids.

### Flow cytometry

To label general exosomal CD63 and TBI exosomal GFAP, 5 μL of PE rat anti-mouse CD63 (BD Biosciences, USA) and 2 μL of Alexa Fluor 647 mouse anti-GFAP (BD Biosciences, USA) were added to 50 μL acoustofluidic-isolated exosome samples and original plasma. After incubation, the samples were diluted with 250 μL PBS and fixed with 4% paraformaldehyde (Santa Cruz Biotechnology, USA). A BD LSR II flow cytometer (BD Bioscience, USA) from the Duke Human Vaccine Institute was used to examine the stained samples. To set the threshold to eliminate noise signals, nonstained, only CD63-stained, and only GFAP-stained samples were also examined. Analysis was performed using BD FACS Diva software and FlowJo V10.2.

## Supplementary information

SupplementalData
