# Supplementary material for: Acoustofluidic separation enables early diagnosis of traumatic brain injury based on circulating exosomes
Source: Microsyst Nanoeng. 2021 Mar 3;7:20. doi: 10.1038/s41378-021-00244-3 (PMC8433131; doi:10.1038/s41378-021-00244-3)
Supplement: Supplementary file 1 — SupplementalData [file 41378_2021_244_MOESM1_ESM.docx]

Figure S1


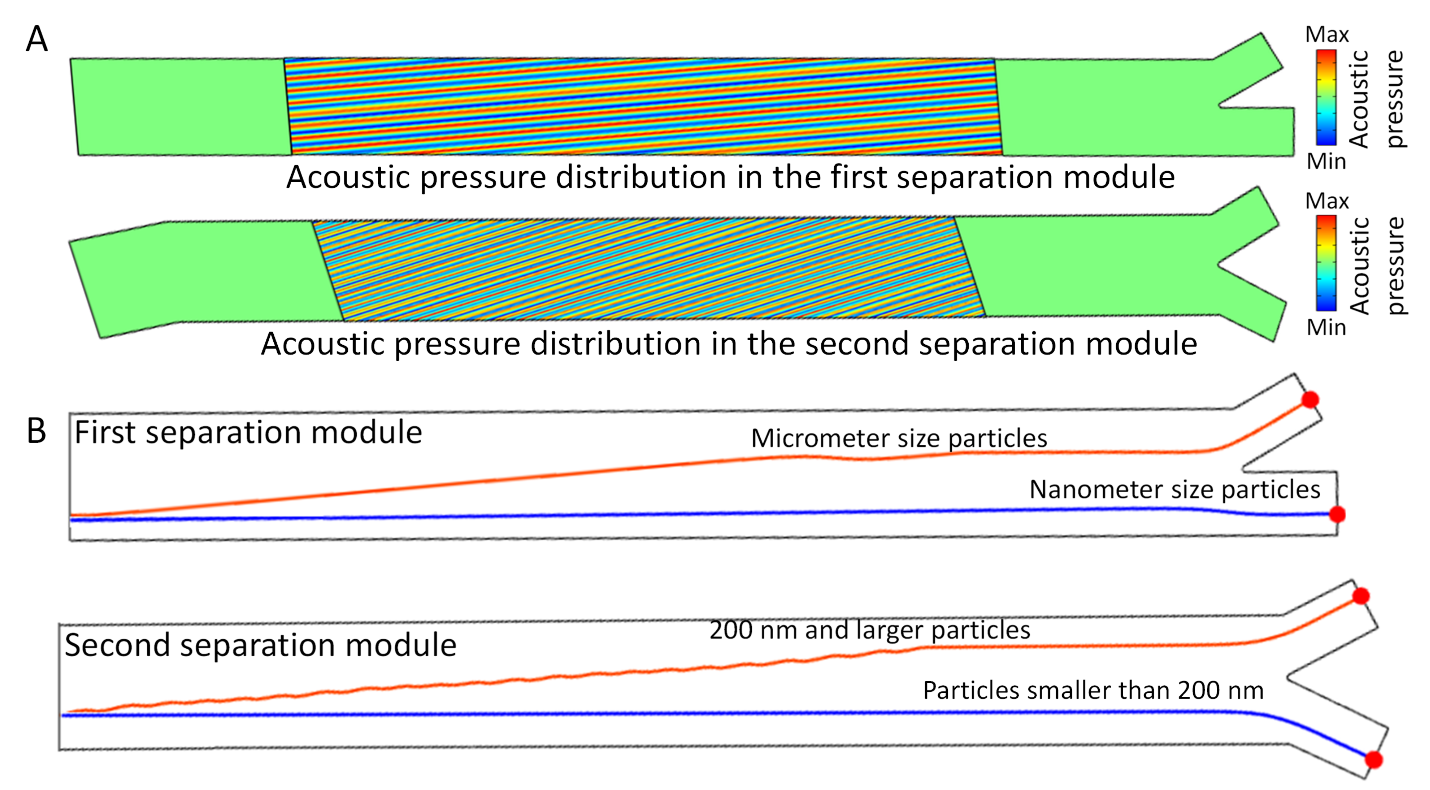


**Fig. S1.** Simulation of (A) acoustic pressure field distribution and (B) particle motion trajectory in the acoustofluidic channel. Continuous pressure nodes generate high acoustic pressure lines and force larger particles, which succumb to larger acoustic radiation forces, move to the upper side of the channel and flow to the waste outlets, while small particles move to the lower side of the channel, and flow to the collection outlet.

Figure S2

**
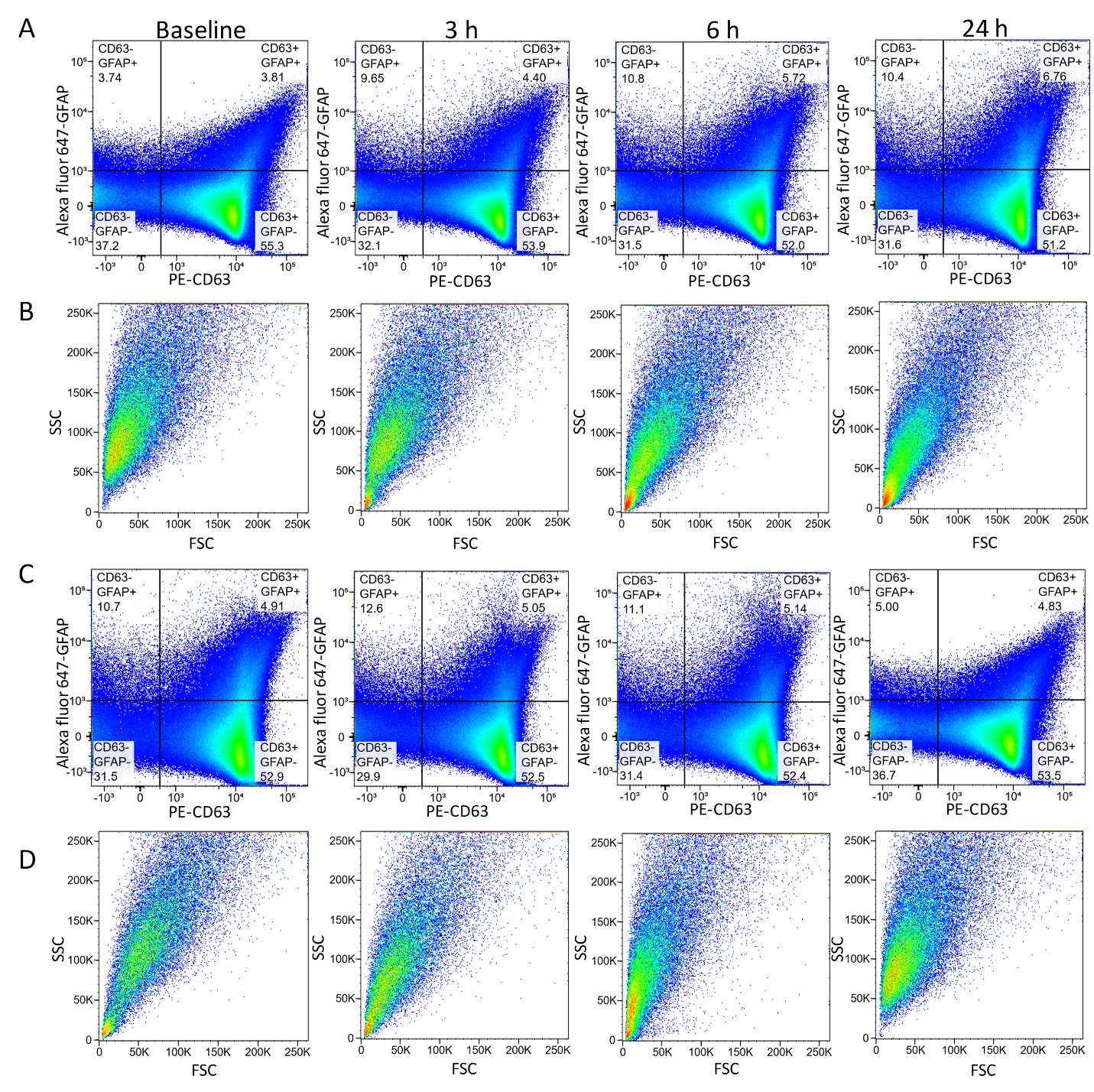
****Fig. S2.** CD63+/GFAP- exosomes population change after TBI treatment of mouse 2. A) Acoustofluidic isolated exosome samples stained by both PE-CD63 and Alexa fluor-647-GFAP collected at different timepoints shows CD63+/GFAP+ events increasing after TBI (the event in CD63+/GFAP+ dimension increased from 3.81% of baseline to 4.40%, 5.72%, and 6.76% in 3, 6, and 24 hour), which indicates an increasing abundance of TBI-exosomes in blood, B) FSC and SSC of CD63+/GFAP+ area for exosomes at each timepoint indicate that the CD63+/GFAP+ events are initially induced by noise from large particles but then induced by exosome signals after TBI, C) direct flow cytometry analysis using plasma samples did not demonstrate CD63+/GFAP+ events increasing after TBI (the event in CD63+/GFAP+ dimension is 4.91%, 5.05%, 5.14%, and 4.83% in baseline, 3, 6, and 24 hour, there is no trend of increasing), D) FSC and SSC of CD63+/GFAP+ area of plasma collected each at various times indicate that most of the signals were noise from large particles.

Figure S3

**
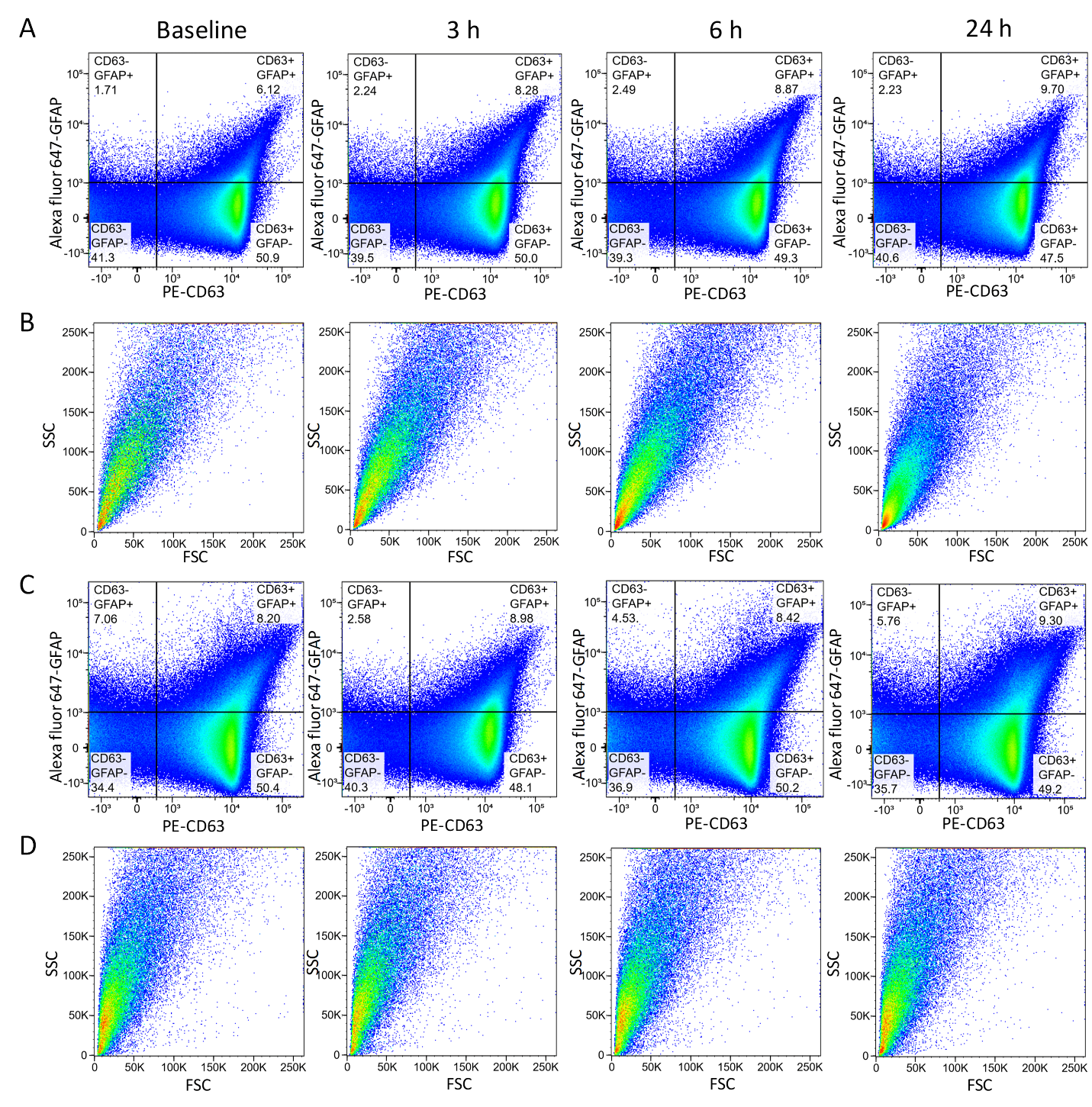
Fig. S3.** CD63+/GFAP- exosome population change after TBI treatment of mouse 3. a) Acoustofluidic isolated exosomes stained by both PE-CD63 and Alexa fluor-647-GFAP, samples collected at different timepoints show CD63+/GFAP+ events increasing after TBI (the event in CD63+/GFAP+ dimension increased from 6.12% of baseline to 8.28%, 8.87%, and 9.70% in 3, 6, and 24 hour), which indicates an abundance of TBI-related exosomes in blood, b) FSC and SSC of CD63+/GFAP+ area for exosomes at each timepoint, which indicates that the CD63+/GFAP+ events were initially induced by noise from large particles, but then induced by exosome signals after TBI, c) direct flow cytometry analysis using plasma samples cannot demonstrate CD63+/GFAP+ events increasing after TBI (the event in CD63+/GFAP+ dimension is 8.20%, 8.98%, 8.42%, and 9.30% in baseline, 3, 6, and 24 hour, there is no trend of increasing), d) FSC and SSC of CD63+/GFAP+ area of plasma collected each timepoint; most of the signals were noise from large particles.
